# Amundsen Sea Mollusca from the BIOPEARL II expedition

**DOI:** 10.3897/zookeys.294.4796

**Published:** 2013-04-22

**Authors:** Camille Moreau, Katrin Linse, Huw Griffiths, David Barnes, Stefanie Kaiser, Adrian Glover, Chester Sands, Jan Strugnell, Peter Enderlein, Paul Geissler

**Affiliations:** 1 British Antarctic Survey (BAS), High Cross Madingley Road, CB3 0ET, Cambridge, United Kingdom; 2 Biocentre Grindel and Zoological Museum Hamburg, Martin-Luther-King-Platz 3, 20146 Hamburg, Germany; 3 Natural History Museum, London, UK; 4 Department of Genetics, La Trobe Institute for Molecular Science, La Trobe University, Bundoora, 3086 Vic., Australia

**Keywords:** Mollusca, Antarctica, Amundsen Sea, Bivalvia, Gastropoda, Scaphopoda, Aplacophora, Monoplacophora, Polyplacophora, BIOPEARL II

## Abstract

Information regarding the molluscs in this dataset is based on the epibenthic sledge (EBS) samples collected during the cruise BIOPEARL II / JR179 RRS James Clark Ross in the austral summer 2008. A total of 35 epibenthic sledge deployments have been performed at five locations in the Amundsen Sea at Pine Island Bay (PIB) and the Amundsen Sea Embayment (ASE) at depths ranging from 476 to 3501m. This presents a unique and important collection for the Antarctic benthic biodiversity assessment as the Amundsen Sea remains one of the least known regions in Antarctica. Indeed the work presented in this dataset is based on the first benthic samples collected with an EBS in the Amundsen Sea. However we assume that the data represented are an underestimation of the real fauna present in the Amundsen Sea. In total 9261 specimens belonging to 6 classes 55 families and 97 morphospecies were collected. The species richness per station varied between 6 and 43. Gastropoda were most species rich 50 species followed by Bivalvia (37), Aplacophora (5), Scaphopoda (3) and one from each of Polyplacophora and Monoplacophora.

## Project details

**Project title:** BIOPEARL II-JR 179 RRS James Clark Ross 2008

**Personnel:** Camille Moreau, Katrin Linse, Huw Griffiths, Peter Enderlein and David Barnes

**Funding:** This study is part of the British Antarctic Survey Polar Science for Planet Earth Programme funded by the Natural Environment Research Council.

**Study extent description:** The study area of this dataset was set in the eastern Amundsen Sea and focused on the continental shelf, upper slope and over-deepened shelf basins of the Amundsen Sea Embayment (ASE) and Pine Island Bay (PIB). This dataset presents species occurrences and species richness of the individual epibenthic sledge (EBS) deployments. PIB appears to be the third largest drainage outlet of the West Antarctic Ice Sheet (Lowe and Anderson 2002). This area was chosen for the BIOPEARL II cruise as it has never been subject to benthic sampling before. Furthermore it shows a unique oceanography over its continental shelf defined by the Antarctic Circumpolar Deep Water (Jenkins et al. 2004). The presence of deep basins and troughs allows the trapping of warm Circumpolar deep Water (3.5°C above the in situ freezing point) on the continental shelf of the ASE and PIB (Jacobs et al. 2011). Vaughan (2008) assumed that these particularly warm waters are one of the reasons of the high melting rate reported at the base of the floating ice shelf in these regions. The seabed of the ASE, which is of particular interest in this benthic work, presents the marks of historic, glaciations and deglaciations, together with icebergs scouring and melt-water channels (Dowdeswell et al. 2006; Nitsche et al. 2007; Larter et al. 2009; Noormets et al. 2009). One of the other characteristics of the area is the perennial sea ice cover (Graham et al. 2010).

**Design description:** The Amundsen Sea is a very under sampled area on the Antarctic continental shelf, according to a recent gap analysis carried out by Griffiths et al. (2011). BIOPEARL (Biodiversity dynamics : phylogeography, evolution and radiation of life in Antarctica), a core project at the British Antarctic Survey, studied the southern Bellingshausen and eastern Amundsen seas to assess the biodiversity at local and regional scales (comparable to the BIOPEARL 2006 cruise to the Scotia Sea) and investigate the phylogenetic relationships of selected marine invertebrate taxa and their biogeography in reference to the climatological, oceanographical and geological history of the Bellingshausen/Amundsen Seas. The results are used to determine of the role of Antarctica and extreme environments in general in evolutionary innovation and generation of global biodiversity. The species presence data are added to SOMBASE (Southern Ocean Mollusc Database www.antarctica.ac.uk/sombase). SOMBASE generated initial core data system upon which SCAR’s Marine Biodiversity Information Network (SCAR-MarBIN) was built. As SCAR-MarBIN is the Antarctic Node of the international OBIS network, the SOMBASE data system was designed to comply with the Darwin Core standards. Regarding the dataset, the existing Data Toolkit from SCAR-MarBIN was used (http://www.scarmarbin.be/documents/SM-FATv1.zip), following the OBIS schema (http://iobis.org/data/schema-and-metadata). The dataset was uploaded in the ANTOBIS database (the geospatial component of SCAR-MarBIN), and the taxonomy was matched against the Register of Antarctic Marine Species, using the Taxon Match tool (http://www.scarmarbin.be/rams.php?p=match). The dataset meets the Darwin Core requirements and was designed around this data schema.

**Figure 1.**
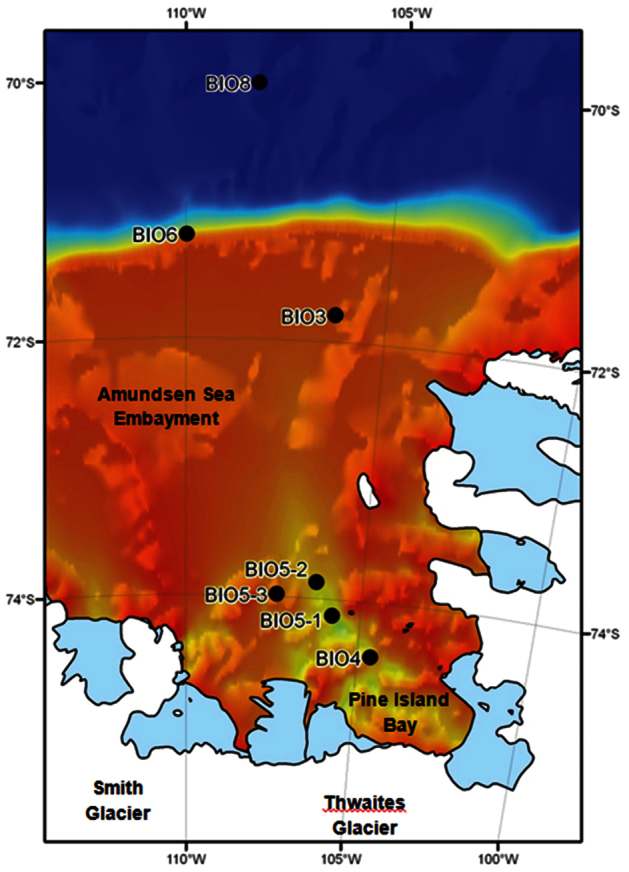
BIOPEARL II EBS stations in the Amundsen Sea and its seafloor topography. The bathymetry colour legend goes from 200m (red) to 4000m (dark blue).

**Figure 2.**
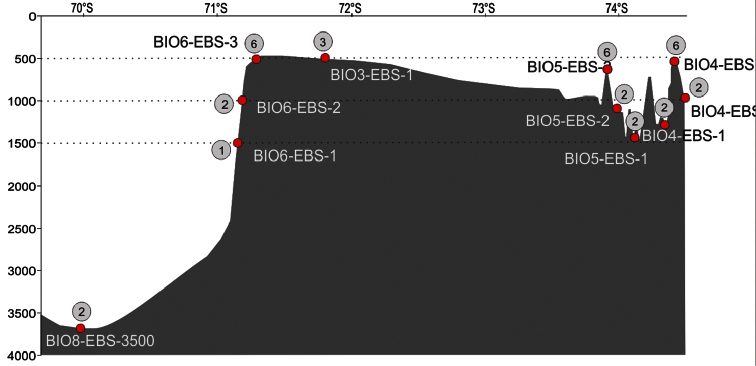
BIOPEARL II station and number of replicates reported on the seafloor topography of the Amundsen Sea Embayment.

**Sampling description:** Five locations in the Pine Island Bay (PIB) and Amundsen Sea Embayment (ASE) at different depths ranging from 476 to 3501m have been sampled using an epibenthic sledge (EBS). Most deployments were made along depth transects from shallow to overdeepened continental shelf and to deeper slope (Figure 1 and 2). At three of the five locations samples were taken at ~500m, ~1000m and ~ 1500m depths, due to the particular geomorphology (presence of deep troughs close to the continent) of the ASE continental plateau. At each site, replicates (individual stations) were taken to assess habitat homogeneity and their number depended on water depth; three to six replicates were taken at 500m and two at 1000m, 1500m and 3500m depth. The BIOPEARL II cruise report is available from the British Oceanographic Data Centre (https://www.bodc.ac.uk/data/information_and_inventories/cruise_inventory/report/8277/).

This dataset presents 34 EBS deployments: 21 of which were performed at a depth of 500m at four different sites (BIO3-1, BIO4-3, BIO5-3 and BIO6-3) , six at a 1000m depth in three areas (BIO4-2, BIO5-2 and BIO6-2), five at a depth of 1500m at three different sites (BIO4-1, BIO5-1 and BIO6-1), and two replicates at site BIO8-3500 in 3500m depth. For three of the five locations, sites were positioned along vertical transects sampling at 500m, 1000m and 1500m with repeat deployments of the EBS. The sites BIO4-1, BIO4-2 and BIO4-3 and BIO6-1, BIO6-2 and BIO6-3 were situated in the same local area; while the sites BIO5-1, BIO5-2 and BIO5-3 were dispersed over a wider area because of ice cover. The EBS consist of on an epi-(below) and a supra-(above) net. Each of these nets has a mesh size of 500μm and an opening of 100x33cm. The cod end of both nets is equipped with net-buckets containing a 300μm mesh window (Brenke 2005). The EBS was trawled for 10 minutes on the sea bed at a 1 knot speed for deployments in 500m to 1500m and for 20 min in 3500m. Following Brenke (2005) that epi- and supra-nets are collecting the same fauna, these were pooled and treated as a single sample.

**Quality control description: **A species name was given to each specimen when it was possible. Individuals not corresponding to described species have been included in the analyses with the family or genus name and a letter or numerical code (e.g. Turbinidae gen. sp.), however they represent a single morphospecies.

For these specimens, further morphological and genetic analyses are necessary to give them a species name but they can be included in this dataset as different species. Finally, specimens too badly damaged for species identification have not been taken in account here.

This dataset presents species occurrences and species richness of the individual EBS deployments.

## Taxonomic coverage

**General taxonomic coverage description: **The present dataset focus on six molluscs classes (Mollusca: Aplacophora, Monoplacophora, Polyplacophora, Gastropoda, Bivalvia & Scaphopoda). It includes respectively for each class:

**Class:** Aplacophora

**Species:** *Aplacophora* sp. 1, *Aplacophora* sp. 2, *Aplacophora* sp. 3, *Aplacophora* sp. 4, *Aplacophora* sp. 5

**Class:** Polyplacophora

**Family:** Leptochitonidae

**Genus:** *Leptochiton*

**Species:** *Leptochiton* sp.

**Class:** Monoplacophora

**Family:** Micropilinidae

**Genus:** *Micropilina*

**Species:** *Micropilina* sp.

**Class:** Gastropoda

**Family:** Scissurellidae, Ataphridae, Mangeliidae, Capulidae, Calliotropidae, Seguenzioidea, Turbinidae, Turridae, Eulimidae, Limacinidae, Eatoniellidae, Cancellarioidea, Naticidae, Rissoidae, Diaphinidae, Fissurellidae, Raphitomidae, Cylichnidae, Lepetidae, Orbitestellidae, Buccinidae, Mathildidae, Newtoniellidae, Marginellidae, Cavoloniidae

**Genus:** *Anatoma*, *Trochaclis*, *Lorabela*, *Belalora*, *Torellia*, *Capulus*, *Calliotropis*, *Brookula*, *Brookula*, *Lissotesta*, *Liotella*, *Cirsonella*, *Balcis*, *Onoba*, *Hemiaclis*, *Limacina*, *Eatoniella*, Cancellaridae gen., *Falsilunatia*, *Pseudomauropsis*, *Sinuber*, *Powellisetia*, *Rissoid*, *Toledonia*, Fissurellidae gen., *Cornisepta*, *Zeidora*, *Pleurotomella*, *Cylichna*, *Iothia*, *Microdiscula*, *Pareuthria*, *Turritellopsis*, *Cerithiella*, *Eumetula*, *Marginella*, *Clio*

**Species:** *Anatoma euglypta*, *Trochaclis antarctica*, *Lorabela pelseneeri*, *Belalora* cf *striatula*, *Torellia insignis*, *Capulus* sp., *Calliotropis pelseneeri*, *Brookula* cf *charleenae*, *Brookula* sp. b, *Lissotesta* sp., Liotella sp., *Liotella* cf *endeavourensis*, *Cirsonella* *extrema*, *Turbinid* sp. *Turrid* sp. 1, *Turrid* sp. 2, *Balcis* sp. *Onoba* cf *gelida*, *Hemiaclis incolorata*, *Limacina helicina*, *Eatoniella* cf *kerguelenensis regularis*, *Cancellarid* sp. *Falsilunatia* sp. *Pseudomauropsis anderssoni*, *Sinuber microstriatum*, *Powellisetia* cf *deserta*, *Rissoid* sp. *Toledonia* sp. 1, *Toledonia* sp. 2, *Toledonia* cf *elata*, Fissurellidae gen. sp. 1, Fisserulidae gen. sp. 2, *Cornisepta antarctica*, *Zeidora antarctica*, *Pleurotomella* cf *simillima*, *Cylichna* sp. *Iothia* sp. *Microdiscula* sp. *Pareuthria* cf *innocens*, *Turritellopsis gratissima*, *Cerithiella* cf *lineata*, *Cerithiella* cf *erecta*, *Eumetula* cf *strebeli*, *Marginella ealesae*, *Clio antarctica*

**Class:** Bivalvia

**Family:** Nuculanidae, Nuculidae, Yoldiidae, Limopsidae, Philobryidae, Mytilidae, Limidae, Pectinidae, Propeamussiidae, Thyasiridae, Motacutidae, Lasaeidae, Cyamiidae, Carditidae, Thraciidae, Cuspidariidae, Lyonsiidae, Poromyidae, Neoleptonidae, Siliculidae, Arcidae, Vesicomyidae, Tindariidae, Bathyspinulidae

**Genus:** *Propeleda*, *Ennucula*, *Yoldiella*, *Limopsis*, *Philobrya*, *Adacnarca*, *Dacrydium*, *Limatula*, *Adamussium*, *Hyalopecten*, *Cyclochlamys*, *Thyasira*, *Mysella*, *Waldo*, *Cyamiocardium*, *Cyclocardia*, *Thracia*, *Cuspidaria*, *Myonera*, *Lyonsia*, *Poromya*, *Neolepton*, *Silicula*, *Bathyarca*, *Vesicomya*, *Tindaria*, *Bathyspinula*

**Species:** *Propeleda longicaudata*, *Ennucula* sp. *Yoldiella ecaudata*, *Yoldiella sabrina*, *Yoldiella valettei*, *Yoldiella* cf *profundorum*, *Yoldiella oblonga*, *Yoldiella* sp. *Limopsis longipilosa*, *Limopsis knudseni*, *Philobrya sublaevis*, *Philobrya quadrata*, *Adacnarca nitens*, *Dacrydium albidum*, *Limatula Limatula* sp. *Limatula Antarctolima* sp. *Adamussium colbecki*, *Hyalopecten pudicus*, *Cyclochlamys pteriola*, *Cyclochlamys gaussiana*, *Thyasira* sp. *Mysella antarctica*, *Waldo* sp. *Cyamiocardium denticulatum*, *Cyclocardia astartoides*, *Thracia meridionalis*, *Cuspidaria infelix*, *Cuspidaria minima*, *Myonera fragilissima*, *Lyonsia arcaeformis*, *Poromya antarctica*, *Neolepton* sp. *Silicula rouchi*, *Bathyarca sinuata*, *Vesicomya sirenkoi*, *Tindaria* sp. *Bathyspinula* sp.

**Class:** Scaphopoda

**Family:** Dentaliidae, Pulsellidae, Gadilidae

**Genus:** *Dentalium*, *Striopulsellum*, *Cadulus*

**Species:** *Dentalium majorinum*, *Striopulsellum minimum*, *Cadulus thielei*

## Spatial coverage

**General spatial coverage: **Amundsen Sea, Antarctica

**Coordinates: **74°29'24"S and 70°1'12"S; 110°5'24"W and 104°20'24"W

**Temporal coverage: **February 18, 2008–April 11, 2008

**Natural collections description**

**Parent collection identifier: **British Antarctic Survey

**Collection name: **BIOPEARL II EBS Molluscs

**Collection identifier: **Moreau/Linse

**Specimen preservation method: **Ethanol/Formaldehyde

## Methods

### Method step description:

- Epibenthic sledge sampling in the Amundsen Sea

- Once on the deck, the content of the samplers from the first deployment was immediately fixed in 96% undenaturated and pre-cooled (at -20°C) ethanol (Linse 2008) and kept for a minimum of 48 hours in a -20°C freezer and the samplers from the second deployment were fixed in 4 % buffered formalin. If six EBS deployments were carried out at a station, four were fixed in ethanol and two in formaldehyde. Afterwards, these samples were washed in cold sea water and transferred to 80 % ethanol. The treatment in formalin allows cytological studies.

- The taxonomic identification was performed in the British Antarctic Survey laboratory using a stereomicroscope.

## Datasets

### Dataset description

**Object name: **Darwin Core Archive amundsen_sea_molluscs

**Character encoding: **UTF-8

**Format name: **Darwin Core Archive format

**Format version: **1.0

**Distribution: **http://ipt.biodiversity.aq/archive.do?r=amundsenseamolluscs_biopearl_ii

**Publication date of data: **2013-01-09

**Language: **English

**Metadata language: **English

**Date of metadata creation: **2013-01-09

**Hierarchy level: **Dataset
